# Withdrawal of Dr. James B. Hodgkin

**Published:** 1883-01

**Authors:** 


*Withdrawal of Dr. James B. Hodgkin.*—We are requested by Dr. Hodgkin to withdraw his name from the editorial department of this JOURNAL: which is done by the mutual consent of all parties concerned. We take this occasion to thank Dr. H. for his exertion in the interest of the JOURNAL while acting as associate editor, and we wish him every success.

SNOWDEN & COWMAN, *Publishers.*

